# Oscillometry Longitudinal Data on COVID-19 Acute Respiratory Syndrome Treated with Non-Invasive Respiratory Support

**DOI:** 10.3390/jcm13071868

**Published:** 2024-03-24

**Authors:** Chiara Torregiani, Elisa Baratella, Antonio Segalotti, Barbara Ruaro, Francesco Salton, Paola Confalonieri, Stefano Tavano, Giulia Lapadula, Chiara Bozzi, Marco Confalonieri, Raffaele L. Dellaca’, Chiara Veneroni

**Affiliations:** 1 Pulmonology Unit, Department of Medical Surgical and Health Sciences, University of Trieste, Hospital of Cattinara, 34149 Trieste, Italy; 2 Radiology Unit, Department of Medical Surgical and Health Sciences, University Hospital of Cattinara, 34149 Trieste, Italy; 3 Department of Electronics, Information and Biomedical Engineering (DEIB), TechRes Lab, Politecnico di Milano University, 20122 Milano, Italy; raffaele.dellaca@polimi.it (R.L.D.); chiara.veneroni@polimi.it (C.V.)

**Keywords:** COVID-19 ARDS, oscillometry, non-invasive oxygen support, lung mechanics

## Abstract

**Background**: Oscillometry allows for the non-invasive measurements of lung mechanics. In COVID-19 ARDS patients treated with Non-Invasive Oxygen Support (NI-OS), we aimed to (1) observe lung mechanics at the patients’ admission and their subsequent changes, (2) compare lung mechanics with clinical and imaging data, and (3) evaluate whether lung mechanics helps to predict clinical outcomes. **Methods**: We retrospectively analyzed the data from 37 consecutive patients with moderate–severe COVID-19 ARDS. Oscillometry was performed on their 1st, 4th, and 7th day of hospitalization. Resistance (R5), reactance (X5), within-breath reactance changes (ΔX5), and the frequency dependence of the resistance (R5–R19) were considered. Twenty-seven patients underwent computed tomographic pulmonary angiography (CTPA): collapsed, poorly aerated, and normally inflated areas were quantified. Adverse outcomes were defined as intubation or death. **Results**: Thirty-two patients were included in this study. At the first measurement, only 44% of them had an abnormal R5 or X5. In total, 23 patients had measurements performed on their 3rd day and 7 on their 7th day of hospitalization. In general, their R5, R5–R19, and ΔX decreased with time, while their X5 increased. Collapsed areas on the CTPA correlated with the X5 z-score (ρ = −0.38; *p* = 0.046), while poorly aerated areas did not. Seven patients had adverse outcomes but did not present different oscillometry parameters on their 1st day of hospitalization. **Conclusions**: Our study confirms the feasibility of oscillometry in critically ill patients with COVID-19 pneumonia undergoing NI-OS. The X5 z-scores indicates collapsed but not poorly aerated lung areas in COVID-19 pneumonia. Our data, which show a severe impairment of gas exchange despite normal reactance in most patients with COVID-19 ARDS, support the hypothesis of a composite COVID-19 ARDS physiopathology.

## 1. Introduction

Over the last three years, critical COVID-19 pneumonia has been a unique model in which non-invasive oxygen support (NI-OS) was prioritized as the first-line treatment for severe acute respiratory failure. This decision was influenced by factors such as resource paucity [1,2,3] and concerns regarding the initially poor prognosis data of invasive mechanical ventilation [4,5]. NI-OS improved the survival in patients with critical COVID-19 pneumonia [6,7]. Tools for the prompt recognition of non-invasive ventilation (NIV) failure have been extensively sought from the beginning of the pandemic to limit the potential detrimental effects of NIV failure on patient survival [1,7,8].

Lung compliance in COVID-19 ARDS has been a matter of debate. Even if COVID-19 ARDS patients present a low lung compliance similar to typical ARDS patients [9,10,11,12], with the worst compliance values related to adverse outcomes [9], a subgroup of COVID-19 ARDS patients with higher lung compliance values has been demonstrated since the beginning of the pandemic [13,14]. Higher compliance was initially reported to be linked to a better prognosis if it did not deteriorate in a low-compliance phenotype [15]. However, this observation was not confirmed in other studies [11,16,17].

Oscillometry, also known as the Forced Oscillation Technique, measures the resistance (Rrs) and the reactance (Xrs) of the respiratory system [18,19]. Rrs is related to the resistive properties of the respiratory system: a lower Rrs corresponds to lower pressure needed to overcome the resistive load for a given respiratory flow. Xrs is linked to the elastic and inertial properties of the respiratory system. In particular, the Xrs increases with increasing lung compliance [20]. Studies in animal models of ARDS and preterm infants with respiratory distress syndrome showed that Xrs provides insights about respiratory compliance and lung volume recruitability [19,21,22]. In particular, in the animal model of ARDS, Xrs was strictly related to the collapsed lung areas identified by CT scan [19]. 

We have previously shown that an oscillometry measurement is feasible in the specific setting of COVID-19 ARDS patients undergoing NIV cycles [23]. We wondered whether COVID-19 ARDS patients with extended consolidations in their Computer Tomography (CT) had lower respiratory compliance, as estimated by Xrs, and whether a higher intubation risk was related to worsening respiratory mechanics. Therefore, we aimed to (i) observe lung mechanics at patients’ admission to the Respiratory Intensive Care Unit (RICU) and their changes during their residence in the RICU, (ii) compare lung mechanics with clinical and CT data, and (iii) evaluate whether lung mechanics together with clinical and imaging data could predict clinical outcomes. 

## 2. Materials and Methods

### 2.1. Study Population

We analyzed the data from 37 consecutive patients hospitalized at the Respiratory Intensive Care Unit (RICU) COVID-19 of the Pulmonology Ward of the Azienda Sanitaria Universitaria Giuliano Isontina (Trieste, Italy) between the 5 April and the 5 May 2021. The local Ethics Review Board approved the protocol (CEUR Friuli Venezia Giulia ID: 3951) and waived informed consent due to the retrospective nature of this study. 

All the patients underwent NIV because of their inability to maintain a SaO_2_ > 92% despite optimized high-flow oxygen support. Inclusion criteria were a confirmed diagnosis of SARS-CoV-2 infection through a nasopharyngeal swab; moderate–severe respiratory failure, defined as PaO_2_/FiO_2_ ≤ 200 in the course of a high-flow nasal cannula (HFNC) with at least a 50 L/min airflow; and hemodynamic stability. Exclusion criteria were a Glasgow Coma Scale (GCM) score < 15, cognitive impairment, severe respiratory and cardiac comorbidities, and age < 18. All patients immediately underwent infusion of corticosteroids, and cycling of prone positioning was applied if tolerated. NIV was delivered using an oro-nasal mask. 

A positive end-expiratory pressure (PEEP) of at least 8 cmH2O was titrated to the patient’s tolerance, verifying the absence of signs of intrinsic PEEP on the ventilator monitor.

Pressure support of at least 3 cmH_2_O was titrated to achieve an oxygen saturation ≥ 92%. NIV was maintained for as long as tolerated with initial short breaks for oral therapy, nutrition, and hydration, during which HFNC was applied. HFNC was delivered through a heated humidifier (Airvo-2, Fisher and Paykel Healthcare, Auckland, New Zealand) and applied through large-bore binasal prongs with a gas flow rate between 50 and 60 L/min. As gas exchange improved, HFNC periods were increased according to the patient’s tolerance to progressively reduce and subsequently stop NIV cycles. 

Failure of NIV was defined by (i) worsened or unchanged dyspnea and lack of oxygenation improvement; (ii) appearance of signs of muscle fatigue, unmanageable tracheal secretions, or complete intolerance to the device; (iii) development of hemodynamic instability and deterioration of cognitive status. 

### 2.2. Data Collection

On admission, the PaO_2_/FiO_2_ ratio in the course of an HFNC was calculated. Laboratory and clinical data were reported within the first 24 h. In particular, C Reactive Protein (CRP) and D-dimer were selected as laboratory biomarkers because of their strong prognostic value [24,25,26]. Moreover, lactate dehydrogenase (LDH) was selected because of its relationship to lung tissue damage [26]. Comorbidities were assessed using the Charlson Comorbidity Index (CCI), and the severity of the patient’s presentation was calculated using the APACHE II score. Within one week of RICU hospitalization, a computed tomographic pulmonary angiography (CTPA) scan was performed because of a clinical suspicion of pulmonary embolism in a subset of patients. The duration of their entire hospitalization and RICU stay were recorded. 

### 2.3. Measurements

Oscillometry was applied during HFNC periods, briefly suspending HFNC treatment for the time of the measurement. Measurements were performed at three points in time: within 24 h after admission to the RICU and afterward on the 4th and 7th day of their stay in RICU, if the patient was not transferred. The oscillometry measurements are described in detail elsewhere [24]. Briefly, in each session, triplicate measurements were performed (Resmon PRO Full, ResTech srl, Milano, Italy) in a sitting position following the technical standard [20]. Each measurement lasted less than a minute, and oxygen saturation and ECG were monitored continuously. 

### 2.4. Data Analysis

Measurements presenting a coefficient of variation of R5 (CoV_R5) > 10% were excluded from the analysis, as in the technical standard [20]. We considered in our analysis the following parameters provided by the machine: Rrs and Xrs at 5 Hz (R5 and X5); their inspiratory (R5insp and X5insp) and expiratory (R5exp and X5exp) components; ΔX5, computed as X5insp-X5exp; and the frequency dependence of the resistance computed as the difference between R5 and Rrs at 19 Hz (R5–R19). Z-scores were computed by the machine according to Oostveen et al. [27]. Rrs and Xrs were classified as normal if their z-scores were <1.64 and >−1.64, respectively.

Quantitative CTPA analysis was performed (Lung CT Analyzer, 2.59-3D Slicer). Collapsed (between +99 and −100 Hounsfield Units, HU), poorly aerated, or ground-glass (−101 and −500 HU), normally inflated (−501 and −900 HU), and overaerated (−901 and −1024 HU) areas were calculated as volumes in ml and as percentages (Collapsed mL, Collapsed %, Poorly Aerated ml, Poorly Aerated %, Inflated mL, Inflated %, Overaerated ml, Overaerated %).

Adverse clinical outcomes were defined as the necessity of endotracheal intubation or death in those subjects for whom NIV was the ceiling treatment. A favorable clinical outcome was defined as improvement with discharge to the medical ward. The days of patients’ RICU stay and total hospital stay (including days in rehabilitation wards) were considered as additional outcomes. 

### 2.5. Statistical Analysis

Correlations between oscillometry, laboratory, and CTPA data were evaluated by Spearman’s rank correlations. The Mann–Whitney rank sum test tested the differences between patients with favorable and adverse outcomes. A linear mixed model, which considered fixed effects for the intercept and time plus a random effect for the intercept for each patient, tested the changes in oscillometry parameters with time. In the subgroup of favorable outcomes, correlations between length of hospital stay and patient data were tested by Spearman’s rank test. The data were analyzed using Matlab R2020b (MathWorks, Natick, MA, USA).

## 3. Results

Thirty-seven consecutive patients underwent oscillometry measurements within 24 hours of their admission to the RICU. All our patients met the new definition of non-intubated ARDS [28]. All patients tolerated these measurements. One patient was excluded from the study because he was affected by severe COPD. Four patients presented measurementss with a CoVRrs > 10% and were excluded. 

**Figure 1:**
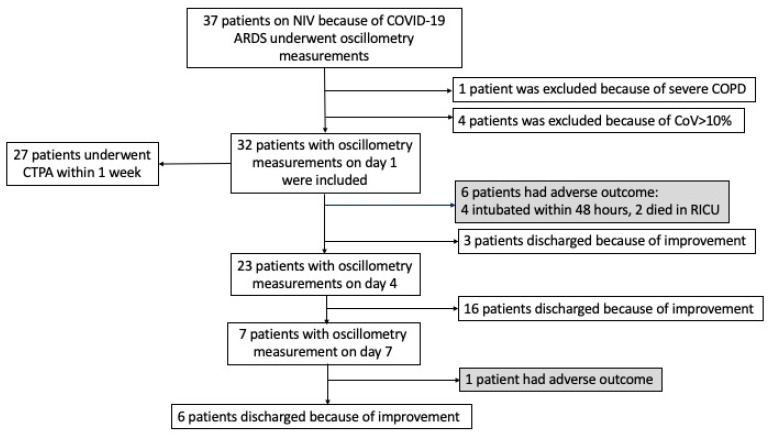
Flowchart of the patients’ cohort.

Twenty-five patients (78.1% of those included) improved and were discharged to medical wards. Seven patients (21.9%) showed an adverse outcome, with five patients undergoing endotracheal intubation. Four of these patients were intubated within two days of their entrance to the RICU, and one patient on the 9th day of their RICU stay, because of worsening respiratory conditions. Two patients who were not suitable for intubation died in the RICU after 8 and 23 days, respectively, and both underwent oscillometry measurements only on the first day because of their severe condition. Two of the five intubated patients died during Intensive Care Unit hospitalization. Figure 1 shows the flowchart of the studied subjects, and Table 1 summarizes their characteristics.

### 3.1. Lung Mechanics

**Figure 2:**
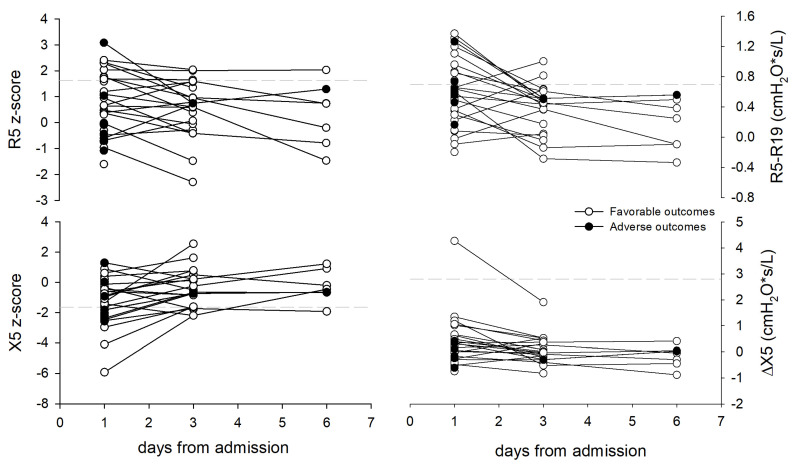
(**Left panel**): Changes in R5 and X5 z-score with time in all the studied subjects. Dashed lines represent the R5’s upper limit of normality and X5’s lower limit of normality. (**Right panel**): Changes in R5–R19 and ΔX5 with time in all the studied subjects. Dashed lines indicate previously proposed upper thresholds for small-airway dysfunction onR5–R19 graph [29] and for EFL_T_ on ΔX5 graph [30].

At the first oscillometry measurement, 11 patients had an abnormal X5 (34%), and 8 patients had an abnormal R5 (25%), for a total of 14 patients with an abnormal R5 or X5. Twenty-one patients underwent oscillometry on their 4th day, and 7 on their 7th day of hospitalization. In general, their R5 (*p* < 0.001), R5–R19 (*p* = 0.0001), and ΔX (*p* = 0.01) decreased with time, while their X5 increased (*p* = 0.001) (Figure 2). 

Only one patient presented ΔX5 values below the tidal expiratory flow limitation threshold. According to a previous spirometry, this patient was the only one affected by COPD with a mild airflow limitation.

### 3.2. Comparison with Imaging and Clinical Data

CTPA was available for 28 patients (Table 1). A pulmonary embolism was found in three patients. The median time from RICU hospitalization to CTPA execution was 0 days (IQR 2 days). All the patients with adverse outcomes had a CTPA performed. The volume of collapsed tissues varied between 293 mL and 1076 mL, while the poorly aerated tissue volume varied between 666 mL and 1905 mL (Table 1). The R5 and X5 z-scores weakly correlated with normal and collapsed tissue (Table 2). The number of collapsed areas and normal parenchyma also correlated with the clinical data (Table 2). 

The seven patients with adverse outcomes had a worse PaO_2_/FiO_2_, higher LDH, higher HACOR index, less normal parenchyma at their CTPA, and longer time interval to their RICU stay, but no different oscillometry parameters (Table 1). Four of these seven patients had abnormal oscillometry values; three died and one underwent intubation. A total of four patients died in our cohort. Three of them had an abnormal initial X5 and died or were intubated within the first 3 days, while the other one, with a normal X5, died in the RICU after 23 days of hospitalization.

### 3.3. Relationship with Clinical Outcomes 

It was not possible to assess the changes in lung mechanics over time in relation to clinical outcomes, as only one patient with adverse outcomes had longitudinal measurements taken. The only variable able to discriminate between favorable and adverse outcomes in the multivariate analysis was the HACOR index (*p* = 0.01). The length of hospitalization in patients with favorable outcomes correlated only with their clinical data (Table 3) and not with their oscillometry parameters.

## 4. Discussion

Our data showed that lung mechanics were normal in the majority of COVID-19 ARDS patients needing NI-OS, and that they generally improved further during hospital stay. Their X5 values at hospital admittance correlated with the collapsed areas on their CT scan, but not with poorly aerated areas (ground-glass), and did not discriminate the patients with that would have adverse outcomes. 

Our data confirmed our recent exploratory data on oscillometry’s feasibility in critical COVID-19 pneumonia and its capability to track resistance and reactance changes during patients’ hospital stays [23]. Oscillometry has been widely tested in intubated patients with acute severe respiratory failure [31], while data in acute spontaneously breathing patients are scarce [23,32]. To our knowledge, our group applied oscillometry for the first time in the setting of severe acute respiratory failure that needed NIV and an HFNC. This technique proved to be feasible and capable of tracking the changes in lung mechanics in COVID-19 ARDS treated non-invasively. The condition of “silent hypoxia”, due to the relatively low initial work of breathing [33,34], could have favored the application of oscillometry in these patients. The feasibility of oscillometry patients with other forms of severe acute respiratory failure undergoing NI-OS should be tested. Oscillometry’s feasibility in this context may enable lung function tests in patients with a limited capability of performing spirometry [35].

### 4.1. Lung Mechanics

This longitudinal assessment of lung mechanics was conducted on 23 subjects, including a single subject presenting an adverse outcome 23 days after his hospitalization. In this group, lung mechanics improved over time, showing a significant R5 reduction, X5 increase, and R5–R19 decrease, indicating a progression toward a more homogeneous distribution of time constants in the lung. The ΔX allowed the identification of a patient with a tidal airflow limitation at his first measurement and testified to his improvement on his 3rd day of hospitalization. The reactance increased over time as the dynamic compliance measured by Tonelli et al. in ARDS patients was successfully managed using NIV [36]. Three previous studies applied oscillometry to longitudinally monitor the lung mechanics in COVID-19 patients across different settings [23,37,38]. The first study included hospitalized patients in their last days before RICU discharge, the second study reported weekly changes in oscillometry parameters in a patient before, during, and after a severe COVID-19 infection, and the third one reported oscillometry data 3 and 5 months after COVID-19 infections. While all the studies reported lung mechanics improvements over time, differences in the study designs, timing of the measurements, and the use of steroids, which may impact lung mechanics [39,40], prevent a direct comparison of these results. 

### 4.2. Comparison with Imaging and Clinical Data 

Our data confirmed seriously impaired gas exchange without a severely reduced X5, as 68% of our patients had a normal X5. This aligns with the COVID-19 ARDS patients treated with NIV presenting higher dynamic compliance versus typical ARDS and a mismatch between lung mechanics and oxygenation [41].

The CT scan data showed small percentages of collapsed tissue, consistent with previous findings in COVID-19 patients undergoing NI-OS [42] and different types of oxygen support [43,44,45]. A higher percentage of collapsed tissue was reported in intubated COVID-19 ARDS patients [46], but it was still lower than the percentage reported in non-COVID-19 ARDS patients treated with invasive ventilation [47]. In fact, the CT scans of COVID-19 ARDS patients showed discrepancies with the those of typical ARDS, resulting in reduced percentages of collapsed tissue, a higher amount of lung gas when matched for PaO_2_/FiO_2_ [14,48], and a peculiar diffusion/perfusion mismatch [42,49]. The poorly aerated areas in our CT scan data related to PaO_2_/FiO_2_, but our collapsed areas did not. The latter lack of relationship was previously reported in COVID-19 ARDS patients treated non-invasively [48]. Despite being a small fraction of the total parenchyma, the amount of collapsed parenchyma correlated with Xrs. Conversely, ground-glass (poorly aerated areas) areas did not correlate with Xrs, as is consistent with previously reported compliance in severe COVID-19 pneumonia [46]. Interestingly, our oscillometry data were not related to clinical or laboratory data. Reactance did not correlate with PaO_2_/FiO_2_, similar to compliance in a cohort of forty COVID-19 ARDS patients undergoing helmet CPAP [48].

### 4.3. Relationship with Clinical Outcomes

We did not find a difference in initial reactance in patients with adverse outcomes, even if it was abnormal in most patients who died. Consistently, our patients’ collapsed areas were not associated with their outcomes. Aerated areas were related to hospital stays in line with the prognostic value of CT scans in COVID-19 pneumonia [44,45,50,51]. Our results lead towards a major role of perfusion alterations in COVID-19 ARDS [14,42,49,52,53], as Shi et al. [54] showed similar compliance despite a worse gas exchange, higher extra-vascular lung water, and pulmonary vascular permeability index in COVID-19 ARDS versus classical ARDS. Recently, the diffuse pulmonary vascular process and the overperfusion of nonventilated areas were indicated as the two main theories behind the low perfusion/ventilation mismatch in COVID-19 [53]. Also, the poor correlation between hypoxemia and compliance has been underscored [53].

As ancillary data, our results confirmed the prognostic role of the HACOR index [55], easily calculable bedside, in the risk of NI-OS failure [56]. The HACOR index, in our data, was significantly related to poorly aerated tissue for the first time, to our knowledge. Moreover, we confirmed the prognostic values of the severity of the patient’s presentation (APACHE II score) and laboratory indexes (LDH), as proven previously [57]. 

### 4.4. Limitations

Our study has limitations. First, the small number of included subjects, especially in the adverse-outcome group, limited the power of this analysis, and our results on their clinical outcomes must be considered exploratory. Second, we could not evaluate the relationship between the longitudinal changes in lung mechanics and outcomes, as only one patient presenting adverse outcomes had multiple measurements taken. Third, our CTPA data were not gathered at the same time as the oscillometry data. However, they were performed within a narrow range of days. Fourth, although the data were consecutively collected, this is a retrospective study including a limited number of subjects.

## 5. Conclusions

Our study confirms the feasibility of oscillometry for monitoring improvements in the lung mechanics of critically ill patients with COVID-19 pneumonia undergoing NI-OS. Our data, showing a severely impaired gas exchange in most patients despite their normal reactance, support the hypothesis of a composite COVID-19 ARDS physiopathology.

## Figures and Tables

**Table 1:** Patients’ characteristics.

	ALL (*n* = 32)	FAVORABLE Outcome (*n* = 25)	ADVERSE Outcome (*n* = 7)
***Patient data***			
Age (years)	60 (53; 69)	58 (52; 65)	69 (61; 78)
Females	7 (22%)	7 (28%)	0
BMI (kg/m^2^)	28.5 (26.0; 32.8)	28.7 (26.2; 31.3)	26.5 (24.6; 37.8)
Smokers (no; ex; yes)	18; 11; 3	13; 9; 3	5; 2; 0
Charlson Comorbidity Index	2 (1; 3)	2 (1; 3)	3 (2.5; 3.5)
***Clinical parameters***			
PaO_2_/FiO_2_—HFNC	91 (74; 115)	92 (77; 115)	73 (61; 116)
PaO_2_/FiO_2_—NIV	151 (146; 191)	162 (150; 197)	144 (84; 149) *
TVE/PBW mL/kg	9.1 (7.9; 10.2)	9.0 (8.1; 10.2)	8.2 (7.7; 10.5)
APACHE	8.5 (6.5; 10.0)	8.0 (6.0; 10.0)	9.0 (7.2; 12.7)
LDH units/L	378 (318; 477)	343 (304; 447)	484 (435; 505) *
D-dimer mcg/L	873 (566; 1143)	865 (488; 1087)	1154 (638; 1691)
CRP mg/L	75.0 (28.5; 99.2)	73.8 (32.2; 97.1)	76.3 (23.6; 105.1)
HACOR	3 (2; 4)	3 (2; 4)	4 (4; 6) *
***CT parameters* ^a^**			
Inflated (%)	59.0 (53.0; 63.5)	61.0 (56.5; 65.0)	54.0 (50.2; 54.7) *
Overaerated (%)	13.8 (12.2; 19.6)	14.0 (12.7; 19.2)	13.6 (8.0; 20.4)
Poorly aerated (%)	18.1 (16.1; 25.8)	17.8 (15.3; 24.1)	23.6 (19.1; 28.4)
Collapsed (%)	4.2 (3.4; 5.9)	4.0 (3.4; 5.6)	5.5 (3.9; 6.4)
***Oscillometry parameters***			
R5 z-score	0.43 (−0.46; 1.64)	0.64 (−0.35; 1.70)	−0.08 (−0.64; 0.77)
X5 z-score	−1.04 (−2.27; −0.06)	−0.98 (−2.12; −0.08)	−1.83 (−2.32; −0.22)
dX	0.13 (−0.25; 0.45)	0.21 (−0.27; 0.64)	−0.17 (−0.23; 0.28)
R5–R19	0.59 (0.33; 0.86)	0.60 (0.28; 0.89)	0.58 (0.49; 0.71)
***Hospital stay***			
Hospital stay before RICU admission (days)	1 (0; 3)	1 (0; 3)	2 (0.5; 8) *
RICU stay (days)	6 (4; 7)	6 (4; 7)	2 (2; 8.5)
Total hospital stay (days)	15 (11; 20)	15 (11; 20)	31 (25; 39)

Continuous variables are reported as median (IQR). Categorical variables are reported as numbers (percentage). * *p* < 0.05 with favorable outcomes. BMI: body mass index; ex-smokers: stopped smoking at least 10 years ago; CCI: Charlson Comorbidity Index; PaO_2_/FiO_2_: ratio between PaO_2_ (arterial partial pressure of oxygen (mmHg)) and FiO_2_ (fraction of inspired oxygen in the course of a high-flow nasal cannula (HFNC)) and in the course of NIV (non-invasive ventilation); TVE/PBW: expiratory tidal volume/predicted body weight (Devine formula); APACHE: Acute Physiologic Assessment and Chronic Health Evaluation; LDH: lactate dehydrogenase, CRP: C-reactive protein; HACOR index: Heart rate, Acidosis (pH), Consciousness (GCS), Oxygenation, and Respiratory rate (26); RICU: Respiratory Intensive Care Unit; total hospitalization: sum of days of hospitalization across all the wards that the patients were admitted to during hospitalization. Normal values are CRP < 5 mg/L, D-dimer < 500 mcg/L, and LDH < 250 units/L. ^a^ Data computed on 28 subjects.

**Table 2:** Spearman correlation ρ (*p*_value) with CT parameters.

	Inflated		Poorly Aerated		Collapsed	
	mL	%	mL	%	mL	%
**R5 z-score**	**0.38 (0.045)**	0.30 (0.12)	−0.10 (0.59)	−0.29 (0.13)	0.14 (0.46)	0.02 (0.90)
**X5 z-score**	0.06 (0.76)	0.09 (0.65)	−0.12 (0.53)	−0.18 (0.35)	**−0.38 (0.046)**	−0.37 (0.051)
**PaO_2_/FiO_2_ NIV**	0.17 (0.41)	0.38 (0.06)	**−0.52 (0.01)**	**−0.56 (0.004)**	−0.21 (0.32)	−0.23 (0.27)
**APACHE**	−0.24 (0.20)	**−0.44 (0.02)**	0.28 (0.14)	0.30 (0.10)	0.36 (0.050)	**0.39 (0.033)**
**LDH**	0.05 (0.79)	**−0.60 (0.0005)**	**0.58 (0.0008)**	**0.47 (0.009)**	**0.48 (0.008)**	0.33 (0.08)
**HACOR**	−0.17 (0.41)	−0.34 (0.10)	**0.50 (0.01)**	**0.56 (0.004)**	0.208 (0.33)	0.27 (0.21)

The 28 subjects with CT data are included in this analysis. PaO_2_: arterial partial pressure of oxygen (mmHg), FiO_2_: fraction of inspired oxygen in the course of a high-flow nasal cannula (HFNC), APACHE: Acute Physiologic Assessment and Chronic Health Evaluation, LDH: lactate dehydrogenase. In bold statistically significant results.

**Table 3:** Spearman ρ (*p*_value) with the length of hospitalization in patients with favorable outcomes (25 subjects).

	Days in RICU	Total Hospital Days
PaO_2_/FiO_2_ HFNC (mmHg)	**−0.45 (0.02)**	−0.37 (0.06)
APACHE	**0.48 (0.01)**	0.26 (0.20)
LDH units/L	**0.45 (0.02)**	**0.42 (0.03)**
CT–Inflated % ^a^	−0.39 (0.08)	**−0.53 (0.01)**

^a^ data computed on 21 subjects. PaO_2_: arterial partial pressure of oxygen (mmHg), FiO_2_: fraction of inspired oxygen in the course of a high-flow nasal cannula (HFNC), APACHE: Acute Physiologic Assessment and Chronic Health Evaluation, LDH: lactate dehydrogenase, CT–inflated %: aerated areas as a percentage of the whole at CT scan. In bold statistically significant results.

## Data Availability

All data are contained within the manuscript.
